# IDENTIFYING CAREGIVERS IN US COMMUNITIES WHO REPORT A LACK OF SUPPORT SERVICES

**DOI:** 10.1093/geroni/igae098.2416

**Published:** 2024-12-31

**Authors:** Greta Kilmer, Akilah Ali, Jenny Walker, Katie Spears

**Affiliations:** Centers for Disease Control and Prevention, Atlanta, Georgia, United States; Centers for Disease Control and Prevention, Atlanta, Georgia, United States; Centers for Disease Control and Prevention, Atlanta, Georgia, United States; Oak Ridge Institute of Science and Education, Georgia, United States

## Abstract

Support services for caregivers are important community health tools that can help address health equity. Many caregivers report support services as unavailable, and understanding the characteristics of these caregivers could help identify who could benefit from greater availability or accessibility. Caregivers were identified in 2023 Summer ConsumerStyles for U.S. adults (n=437) and 2022 Estilos for US Hispanic adults (n=122). Availability and use of seven support services were assessed (out-of-home services, in-home services, food and nutrition services, transportation services, financial planning, caregiver support groups or classes, and caregiver skills-based training). Respondents were asked if each of the services were available for them or their care recipient (yes/no), and if they currently used or would use the service (yes/no). Values were weighted in accordance with the U.S. Current Population Survey proportions. A lack of at least one support service was identified if they reported it not available and they would use the service if available. In 2023, 34.2% of U.S. caregivers overall reported a lack of at least one service, which was significantly associated with caregivers having difficulty paying household expenses (p< 0.001) and having a care recipient with Alzheimer’s disease, dementia, or other cognitive impairment (p< 0.05). In 2022, over half of Hispanic caregivers reported a lack of at least one service (56.7%), which was associated with fewer years living in the U.S. (p< 0.01). Findings suggest that community support services are not always available or accessible, and caregivers with financial difficulties or recent immigration report a particular lack of community-based caregiving support resources.

